# Prothrombin Is Responsible for the Lupus Cofactor Phenomenon in a Patient with Lupus Anticoagulant/Hypoprothrombinemia Syndrome

**DOI:** 10.1055/s-0040-1705091

**Published:** 2020-03-09

**Authors:** Vittorio Pengo, Lorena Zardo, Maria Grazia Cattini, Elisa Bison, Elena Pontara, Sara Altinier, Chunyan Cheng, Gentian Denas

**Affiliations:** 1 Cardiology Clinic, Thrombosis Centre, Department of Cardiac, Thoracic and Vascular Sciences, University of Padua, Padova, Italy; 2 Castelfranco Veneto General Hospital - ULSS 2 Treviso, Treviso, Italy; 3 Department of Laboratory Medicine, University Hospital of Padova, Padova, Italy

**Keywords:** antiphospholipid antibodies, lupus coagulation inhibitor, prothrombin

## Abstract

Lupus anticoagulant is a misnomer as it is commonly associated with thromboembolic events. In few cases, the name retains its literal meaning when it characterizes patients with a bleeding disorder. We describe a patient with lupus anticoagulant, hypoprothrombinemia, and major bleeding (lupus anticoagulant/hypoprothrombinemia syndrome). Immunological studies revealed a huge amount of circulating monoclonal immunoglobulin M lambda (IgMλ) antiphosphatidylserine/prothrombin antibodies (14,400 U/mL). Affinity purified monoclonal antibodies (440 U/mL) prolonged the coagulation time of normal plasma by 12.2 seconds (diluted Russell viper venom time) and 25.5 seconds (silica clotting time). The original patient's plasma mixed 1:1 with normal plasma showed a marked prolongation of coagulation times (lupus cofactor) from a ratio of 2.94 to 5.23 in diluted Russel viper venom time and from 2.30 to 3.00 using the silica clotting time. Human prothrombin added to original patient's plasma caused a marked prolongation of coagulation times in diluted Russell viper venom test thus unequivocally explaining the lupus cofactor phenomenon. In conclusion, we have shown that lupus anticoagulant/hypoprothrombinemia syndrome is attributable to monoclonal IgMλ antibodies directed to phosphatidylserine/prothrombin and that prothrombin is the protein responsible for the observed lupus cofactor phenomenon.

## Introduction

Lupus anticoagulant (LA) indicates the presence of a type of “antiphospholipid antibody” that is frequently, but not always, associated with thromboembolic events. Only occasionally, LA is present in an uncommon bleeding disorder, the LA hypoprothrombinemia syndrome (LA-HPS). 1 2 Twenty-eight cases have been described between 1948 and 1994, 3 but the syndrome is rare and its prevalence is uncertain. 4 Here we describe a case of a patient with LA-HPS with an associated lupus cofactor (LC) phenomenon. In 1959, a lupus patient with LA and hypoprothrombinemia was described by Loeliger. 5 Interestingly, the mixing studies (patient plasma plus normal plasma) prolonged instead of shortening the clotting time of patient's plasma. This phenomenon that increased the inhibitor activity by a normal plasma component was called “lupus cofactor” (LC). 6 7 Loeliger suggested that responsible for the (unknown) “cofactor” could be prothrombin (PT), while others said that LC was driven by β2‐glycoprotein I (β2‐GPI). 8 In this report, we describe a patient with LA-HPS caused by circulating antibodies against PT and prove that prothrombin is responsible for the observed LC phenomenon.

## Materials and Methods

### Coagulation and Immunological Studies

Venous blood was collected in 0.109M sodium citrate 9:1 and double centrifuged at room temperature. Obtained plasma was stored at –80°C until use. All the coagulation tests were performed using the appropriate reagents and the ACL TOP instrumentation (Werfen Group, Milan, Italy). LA was detected according to the International Society of Thrombosis and Haemostasis (ISTH) guidelines. 9 Diluted Russell viper venom time (dRVVT) and silica clotting time (SCT) were performed in three steps (screening, mixing, and confirm) and expressed as ratio of coagulation times of patient's plasma to pooled normal plasma (PNP) for all the procedures. To diagnose the presence of LA avoiding the LC effect, the confirmatory test described was also performed using the original plasma diluted 1:1 with PNP.

Solid phase assays for the detection of antiphospholipid (aPL) antibodies anticardiolipin (aCL), aβ2-GPI, antiprothrombin (aPT), and antiphosphatidylserine/prothrombin (aPS/PT) antibodies were performed as previously described 10 following the recommendations of a recent communication from Scientific and Standardization Committee of the ISTH. 11

### Specific Factor Activity

Factor II, factor V, factor VII, and factor X immuno-depleted deficient plasmas (Werfen Goup, Milan, Italy) were used in combination with prothrombin time reagents to determine specific factor activity in citrated plasma. To evaluate a possible inhibitory effect of antibodies present in the plasma on factor II activity, a Bethesda inhibition titration was performed

### Prothrombin Affinity Column

HighTrap 1 mL column (HiTrap NHS-activated HP, GE Healthcare, Uppsala, Sweden) was washed with 1 mM ice-cold HCl to eliminate the preservative (isopropanol). Eight milligrams of human prothrombin (Enzyme Research, South Bend, Indiana, United States) in 1 mL of coupling buffer (0.2M NaHCO3, 0.5M NaCl, pH 8.3) was injected into the column. After 30 minutes at room temperature, the column was washed six times with coupling buffer and multiple washing alternating ethanolamine buffer (0.5M ethanolamine, 0.5M NaCl pH 8.3) and acetate buffer (0.5M sodium acetate, 0.5M NaCl, pH 4.0) to deactivate any excess group. The column was stored in Tris-buffered saline (20 mM Tris, 150 mM NaCl, pH 7.4) until use. One milliliter of patient's plasma was poured into the column and incubated for 1 hour at room temperature. The column was then washed 10 times with 1 mL of Tris-buffered saline pH 7.4 and bound material eluted with glycine-HCl buffer (0.1 M glycine, NaCL 0.5M, pH 2.8) and dialyzed against Tris-buffered saline with pH 7.4.

### Immunofixation

Plasma immunofixation was performed using antibodies anti-immunoglobulin G (IgG), anti-IgA, anti-IgM, anti-kappa (free and bound light chains), and anti-lambda (free and bound light chains) provided by Sebia (Bagno a Ripoli, Florence, Italy), according to the instructions of the assay (Hydragel 2 IF-BJ [HR]). Immunofixation on 10x immunoaffinity purified material was performed with the same technique used for plasma sample.

### Coagulation Tests Using Affinity Purified IgMλ or Human Prothrombin

LA activity of affinity purified immunoglobulin M lambda (IgMλ) aPS/PT was evaluated by dRVVT and SCT. Fifty microliters of PNP were combined with 100 µL of purified material or Tris-buffered saline with pH 7.4. Following incubation of the mixtures for 30 seconds at 37°C, 50 µL of dRVVT or SCT reagents were added and time recorded. To check for the LC effect of prothrombin in dRVVT, 10 µL of a solution of human prothrombin to get a final concentration of 18, 75, and 150 µg/mL were added to patient's plasma. Ten microliters of Tris-buffered saline served as control.

### Clinical Summary

On May 2018, a 74-year-old male Caucasian patient, who had never bled abnormally, was admitted to hospital for an episode of severe hematuria. The patient was not on anticoagulant therapy. In the preceding days, he had a similar episode that spontaneously resolved. Laboratory tests for liver and renal function as well as blood cell count were normal. Remaining blood examinations were unremarkable except for a prolonged prothrombin time and activated partial thromboplastin time (aPTT). Protein electrophoresis and immune-fixation revealed a monoclonal component IgMλ. The patient was affected by Double-Hit large B Cell Lymphoma stage IVB, a condition resistant to chemotherapy and with poor prognosis. An abdominal computed tomography scan revealed a metastatic invasion of the liver and right kidney. Large para-aortic and mediastinal lymph nodes as well as free fluid in the abdomen were evident. Diagnosis was confirmed by liver biopsy. His performance status was ECOG (Eastern Cooperative Oncology Group), that is, capable of only limited self-care, confined to bed 50% or more of waking hours. After a cycle of chemotherapy, the patients presented an acute renal failure and underwent five hemodialysis sessions, while there was a progressive clinical deterioration in the patient's condition. He died after 2 months from the admission to the hospital.

## Results

### Diagnosis of LA-HPS

As shown in Table 1 , the increased prothrombin time ratio was partially corrected in mixing studies, while the high aPTT ratio further increased. Testing for LA was remarkable as both dRVVT and SCT ratio further increased in mixing studies (LC). When patient's plasma diluted 1:1 (thus providing the cofactor present in normal plasma) was initially used as an original plasma the ratio decreased in mixing studies (no more LC phenomenon). Confirming tests proved the diagnosis of LA (significant reduction in coagulation times). Thrombin time was normal. Testing for single coagulation factors showed normal factor V (90%), VII (70%), and X (75%), while prothrombin level was 4%. A low inhibitory effect on factor II was found: 0.8 Bethesda U/mL.

**Table 1:** Patient's coagulation and immunological studies at hospital admission

Coagulation studies a				
Test	Normal values	Ratio	Mixing	Confirm
Prothrombin time	<1.2	2.34	1.56	–
aPTT	<1.16	1.50	2.06	–
dRVVT	<1.2	2.94	5.23	2.64
SCT	<1.2	2.30	3.00	2.25
Modified dRVVT b	<1.2	4.34	3.92	1.77
Modified SCT b	<1.2	2.60	2.45	1.54
**Immunological studies**				
**Test**		**IgG/IgM** **Normal values**	**IgG**	**IgM**
aCL (GPL/MPL units/mL)		<10/< 8	13	94
aβ2-GPI (U/mL)		<13/< 7	6	47
aPT (U/mL)		<14/< 7	6	56
aPS/PT (U/mL)		<30	117	14,400

Abbreviations: aβ2-GPI, anti-β2-glycoprotein I antibodies; aCL, anticardiolipin antibodies; aPTT, activated partial thromboplastin time; aPS/PT, antiphosphatidylserine/prothrombin antibodies; dRVVT, diluted Russell viper venom time; IgG, immunoglobulin G; SCT, silica clotting time.

a Ratio is obtained dividing patient's coagulation time (PT) in seconds by that of pooled normal plasma (PNP); Mixing is the coagulation time of the 1:1 divided by that of PNP; Confirm is the ratio obtained between patient to PNP coagulation times performed with high aPL concentration.

b Before testing, patient's plasma was diluted 1:1 with normal plasma to avoid the Lupus Cofactor phenomenon and false negative confirming test.

LA, hypoprothrombinemia, and bleeding led to the recognition of LA-HPS.

Solid phase assays for aPL antibodies showed moderate-to-high titer of IgM aCL, IgM aβ2-GPI, and IgM aPT. Surprisingly, patient's plasma contained high titer of IgG and extremely high titer of IgM aPS/PT (14400 U/mL). The value of 14,400 U/mL in aPS/PT enzyme-linked immunosorbent assay (ELISA) was obtained diluting patient's plasma to an extent (1:10,000) to allow optical density to fall into the reference curve.

### Immuno-Affinity Purification of Antiprothrombin Antibodies

**Fig. 1:**
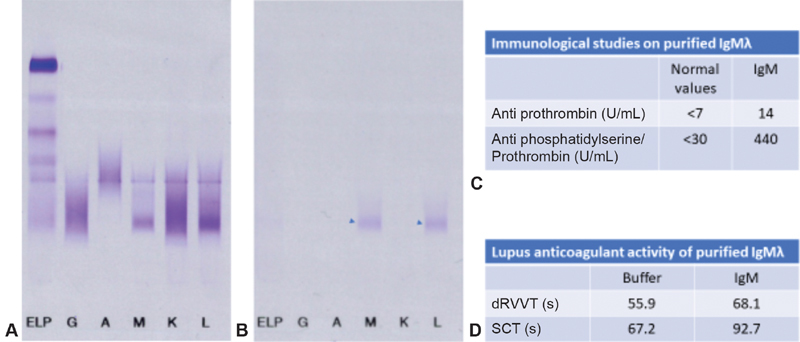
Immunofixation of patient's plasma showing a monoclonal immunoglobulin M lambda (IgMλ) ( **A** ) and its immunoaffinity purification using a prothrombin affinity column ( **B** , arrowheads). Purified material yielded a marked positivity in anti-phosphatidylserine/prothrombin enzyme-linked immunosorbent assay ( **C** ) and possessed lupus anticoagulant activity as shown by diluted Russell viper venom time (dRVVT) and silica clotting time (SCT) ( **D** ).

As shown in Fig. 1 , the material recovered from prothrombin affinity column was a monoclonal IgMλ (34 µg/mL) that showed a marked positivity in IgM aPS/PT ELISA (440 U/mL) and negligible positivity in IgM antiprothrombin ELISA (14 U/mL). Spiking PNP with the eluate containing IgMλ anti PS/PT resulted in marked LA activity in both dRVVT and SCT (12.2 seconds prolongation and 25.5 seconds prolongation compared with buffer, respectively).

### Prothrombin and the Lupus Cofactor Phenomenon

**Fig. 2:**
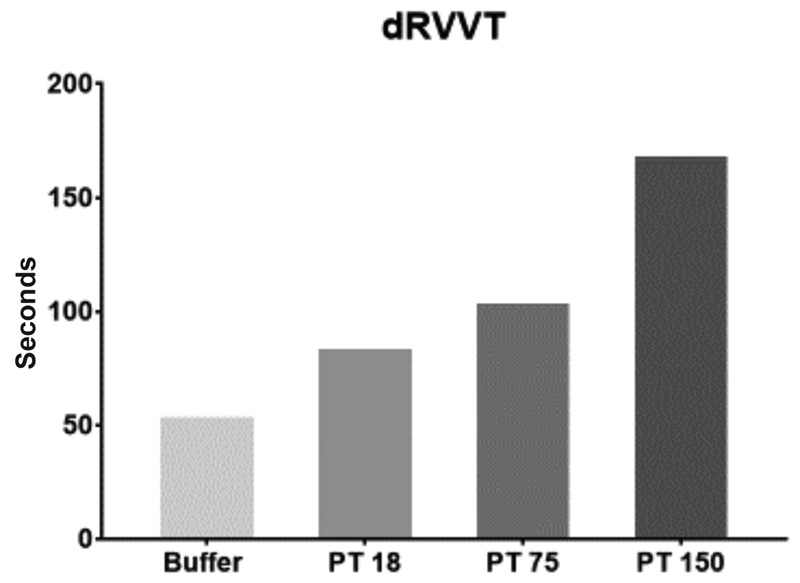
Diluted Russell viper venom time (dRVVT) of patient's plasma without addition of human prothrombin (buffer) and after addition of human prothrombin in increasing amounts (18, 75, 150 µg/mL final concentration in patient's plasma).

To ascertain whether prothrombin was responsible for the LC phenomenon, 10µL of human prothrombin (18, 75, and 150 µg/mL final concentration) was added to patient plasma and results are shown in Fig. 2 . A marked prolongation of coagulation time in a prothrombin concentration manner with respect to buffer was observed in dRVVT.

## Discussion

LA-HPS is a rare acquired disorder caused by prothrombin antibodies. The disease is most common in children and more prevalent in women. Systemic lupus erythematosus and viral infections are the most frequent associated conditions. A recent review of the literature 12 reported that in only 3 of 87 cases the disease was associated with lymphoma. The suspicion of LA-HPS derives from the coexistence of bleeding events with a prolonged aPTT and prothrombin time in combination with a LA. The diagnosis is confirmed by the finding of a reduced prothrombin level. The patient described here showed a strong LA and the LC phenomenon and a huge amount of IgM aPS/PT antibodies. The presence of IgM aCL and IgM aβ2-GPI in the patient's aPL profile might be interpreted as nonspecific binding (false positive results) due to the large amount of circulating IgMλ aPS/PT. We found that anti-PS/PT antibodies were monoclonal IgMλ with LA activity. There was a large discrepancy between the presence of aPS/PT antibodies and those directed toward plain prothrombin (aPT). Indeed, whereas several LA could bind to soluble human prothrombin, 13 14 15 adding PS/phosphatidylcholine vesicles enhanced the extent of the binding. 15 LA antibodies reacting with prothrombin have been postulated to recognize an epitope that becomes exposed only after Ca ^2+^ -mediated binding of prothrombin to phospholipids. 14 16 How we could get the IgMλ anti-PS/PT from a column containing immobilized prothrombin is difficult to comment. One simple explanation might be that the coupling of ligand (prothrombin) through primary amines determines a conformational change of the protein similar to that induced by PS and calcium ions. Another explanation is that the abundant amount of prothrombin (8 mg) used for coupling might increase the local concentration of the antigen without necessarily inducing a radical structural reengagement rearrangement of the protein but increasing the affinity for aPS/PT antibodies. Antiprothrombin antibodies are better identified by Kaolin clotting time, while dRVVT is preferentially sensitive to aβ2-GPI antibodies. 17 In the described patient, both the dRVVT and SCT were equally prolonged suggesting the presence of LA. The confirmatory test showed a modest shortening of coagulation times (dRVVT ratio from 2.90 to 2.64 and SCT ratio from 2.30 to 2.25), a fact that challenged the presence of a LA. Reporting a negative LA without performing mixing tests may thus be misleading 18 and the mixing step is particularly important when a baseline factor deficiency is also present. In fact, upon mixing patient with PNP 1:1, dRVVT and SCT ratios markedly increased showing the presence of a LC. When providing the cofactor by mean of normal plasma (patient plasma mixed 1:1 with normal plasma) in the confirmatory test, a clear reduction in dRVVT and SCT ratios was diagnostic for LA (see the last two rows of Table 1 ). Most likely, the confirmatory test did not work in the original plasma because the very low prothrombin amount limited the rate of reaction despite the increase in phospholipid surface. Prothrombin is a target of aPL antibodies as aPT antibodies are detected in ∼50 to 90% of the patients. 19 On the other hand, aPS/PT antibodies are extremely frequent in patients with LA. 10

The patient suffered from spontaneous bleeding because of very low prothrombin level (4%) and this scenario defines a condition named LA-HPS. Although an association between LA activity and acquired hypoprothrombinemia has been described many years earlier, 1 5 it was not until the early 1980s that the plasma of a patient with the acquired LA-HPS was shown to contain non-neutralizing antiprothrombin antibodies capable of binding to prothrombin in solution thus resulting in rapid clearance of the prothrombin–antibody complex. 20 The very low inhibitory activity found on factor II underlies the presence of mainly non-neutralizing antibodies. Treatment with corticosteroids rapidly increased prothrombin level probably by interrupting the clearance of antigen–antibody complexes. 13

In this study, we have clearly shown for the first time, to the best of our knowledge, that prothrombin is responsible for the observed LC phenomenon. Thus, the insufficient amount of protein impedes the full expression of antibodies inhibitory effect on PL-dependent coagulation tests. Originally, the phenomenon was thought to be due to a patient's plasma being deficient in an undefined cofactor that is essential for LA to exert its anticoagulant effect. The cofactor has been proposed but not proven to be prothrombin or β2‐GPI. 5 8 Although prothrombin is clearly the LC in the patient described here, we cannot exclude that other cofactors may be responsible for the LC phenomenon. However, the role of β2‐GPI as a cofactor can be excluded as the concentration of this protein was normal in the patient's plasma (data not shown). In 1965, Yin and Gaston 6 postulated that the cofactor was a gamma globulin that should be present in correct proportion for the anticoagulant to exert its maximal activity. Furthermore, on the basis of chemical and physical properties, a detailed study by Rivard et al 7 concluded that the cofactor was a complement component with a molecular weight of 200 kD. In our patient, we obtained the LC effect by adding prothrombin to patient's plasma; a minimal amount almost doubled the coagulation time in dRVVT. In conclusion, although these findings are not generalizable, we have shown that in this patient LA-HPS may be attributable to antibodies to aPS/PT and the LC phenomenon is caused by prothrombin.
